# Healing Potential of Propolis in Skin Wounds Evidenced by Clinical Studies

**DOI:** 10.3390/ph15091143

**Published:** 2022-09-14

**Authors:** Cristiano da Rosa, Ian Lucas Bueno, Ana Clara Martins Quaresma, Giovanna Barbarini Longato

**Affiliations:** 1 Stricto Sensu Postgraduate Program in Health Sciences, Universidade São Francisco, Bragança Paulista 12916-900, São Paulo, Brazil; 2 Laboratory of Molecular Pharmacology and Bioactive Compounds, Universidade São Francisco, Bragança Paulista 12916-900, São Paulo, Brazil

**Keywords:** propolis, skin ulcers, clinical studies

## Abstract

Propolis has been used since ancient times for the treatment of skin diseases and, currently, its pharmacological potential for healing and repairing various types of wounds is widely cited in the literature. The healing properties of propolis are mainly attributed to its composition which is rich in phenolic compounds, and propolis has aroused the interest of the pharmaceutical industry as a low-cost product as compared with other treatments and medications; however, most of the published data refer to its effects in vitro and in vivo and, so far, few clinical studies have been carried out proving its therapeutic efficacy. In this article, we aimed to review clinical trail data published in Portuguese, Spanish, and English, in Scielo, PubMed, Google Scholar, Medline, and Lilacs between 1990 and 2021 on the clinical use of propolis for skin ulcers. The potential of propolis as an alternative healing treatment for skin wounds such as diabetic, venous, and surgical wounds, as well as wounds caused by burns, etc., is mainly due to its evidenced properties such as antimicrobial, anti-inflammatory, analgesic, and angiogenesis promoter effects. However, there is a need to standardize the type of administration and the concentration of propolis for each type of wound. Furthermore, further clinical studies are essential to add information about propolis safety and for obtaining the best possible therapeutic benefits from its use.

## 1. Introduction

The occurrence of skin wounds is considered to be a worldwide problem due to high morbidity and treatment costs; in addition to causing negative psychological and social consequences, it directly interferes with the quality of life of those affected [1]. These wounds are characterized by interruptions in the continuity of the skin that can extend from a superficial layer to deeper structures, such as muscles, tendons, and bones [2].

In Brazil, due to an increase in the population’s life expectancy, the prevalence of chronic diseases and conditions has increased, which can lead to the development of skin lesions, such as vascular ulcers and pressure injuries [3].

Epidemiologically, pressure ulcers, diabetic foot ulcers, and venous ulcers are chronic lesions with significant relevance. The occurrence of pressure ulcers is used as an indicator to assess the quality of care provided by health services [4].

Wound healing can occur spontaneously, but when treated, it is considerably faster and presents more satisfactory functional and aesthetic results [5]. The treatment of skin wounds depends on the intrinsic and extrinsic factors of a lesion, which occur dynamically and depend on the evolution of the healing phases. There are currently numerous treatment options on the market. The choice depends on the financial resources of the patient and/or the health unit and they generally try to be adequate for the nature, location, and size of the wound [6].

In open wounds, the old controversy between dry and wet bandages has given way to a current proposal of occlusion and maintenance of the moist environment. Commonly used bandages contain substances such as silver sulfadiazine that has antibacterial action; collagenase enzymatic ointment, for wounds with devitalized tissue; and essential fatty acids, for pressure ulcer prevention or treatment of superficial open wounds with or without infection. There are also bandages that have been developed with well-defined proposals, which have a high cost, based on the use of hydrocolloids, hydrogel, calcium alginate, activated carbon, and hydropolymer adhesive [7].

Among the alternative healing therapies, natural products have been widely used due to their therapeutic properties, availability, and low cost. Among these products, propolis stands out. Since ancient times, propolis has been cited as being used and considered by some people as a medicine for skin diseases. At the end of the 19th century, it was used as a healing agent, and later, it was used in the Second World War in several Soviet clinics [8].

Propolis is a resin-like product that is rich in enzymes, created from the secretions of various plants and mixed with bee saliva. Propolis is composed of 50% resins, 30% waxes, 10% essential oils, 5% pollen, and 5% other organic and mineral compounds [9].

It has been estimated that flavonoids are the main compounds responsible for the beneficial effects of propolis. They are defined as phenolic compounds from plants, which act in different physiological processes, including the absorption of vitamins, the healing process as antioxidants, and exerting the antimicrobial and modulating function of the immune system [10].

Although propolis is a natural product and is currently very commercialized, skin contact can generate allergies due to some of its formulation components, such as caffeic acid esters and cinnamic acid esters, with compound 1-1 dimethylallyl caffeic acid (LB-1) being the most important contact allergen identified in propolis. Therefore, clinical and safety studies related to propolis are still necessary [11]. In view of the above and with the objective of contributing to the development of natural products as alternative healing treatments in skin lesions, in this study, we aimed to investigate the literary data regarding the healing potential of propolis and its therapeutic effects that have been confirmed in clinical studies.

## 2. Results

**Figure 1:**
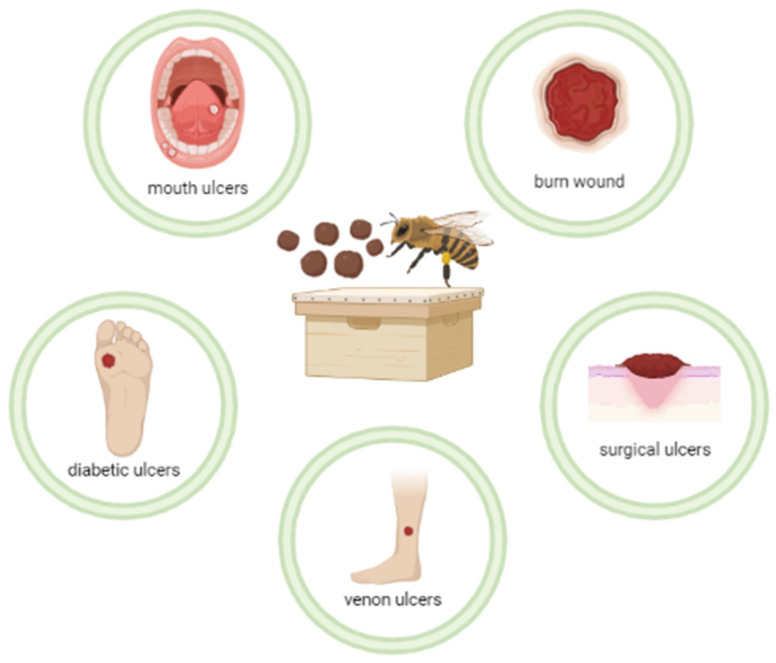
The main ulcers on which propolis was clinically evaluated in the studies described in this work.

Table 1 shows the clinical studies found in the databases, from 1990 to 2021. Twelve articles were included that investigated the healing potential of propolis in formulations such as an ointment, a spray, and a liquid extract. Figure 1 summarizes the types of ulcers that were described in these clinical studies and on which propolis has shown to have a healing action.

## 3. Discussion

### 3.1. Diabetic Ulcer

One of the most prevalent diseases these days is diabetes. This disease affects a large portion of the world population and involves several secondary health problems, such as diabetic foot. It is known that diabetic foot is mainly caused by hyperglycemia, leading to an increase in sorbitol and other compounds, which leads to diabetic macro- and microangiopathy, generating peripheral neuropathy and favoring the appearance of diabetic foot [23]. Therefore, there is a need for topical therapies to reverse this disease of diabetes, and propolis is included among such therapies.

A clinical study, which was conducted in 2019, aimed to evaluate the effect of propolis as an adjuvant product in diabetic foot ulcers, and reported that propolis had healing power and also influenced local inflammation by decreasing some inflammatory cytokines such as TNF-alpha and increasing the expression of anti-inflammatory cytokines such as IL-10 [15].

In this aspect, Afkhamizadeh et. al. [16] analyzed the effect of 5% propolis ointment on human diabetic foot ulcers (Wagner grades 1 and 2) in two groups. The obtained results showed that, although the erythema and exudate in the wound milk were not significantly altered with this treatment, the area of the lesion decreased and a healing process was achieved in 4 weeks of treatment with the topical use of 5% propolis ointment.

Another diabetic foot ulcer was treated with a mixture containing propolis, myrrh and, bee honey (MPH). After debridement of non-viable tissues, the patient was instructed to clean the ulcer, and then to fill the cavity with the MPH paste. After four weeks of treatment, the ulcer healed and the patient returned to work [19].

Another study conducted by Henshaw and colleagues aimed to assess whether propolis ointment was well tolerated in diabetic foot ulcers. They concluded that, after 1 to 3 weeks of treatment, it was possible to observe improvement in healing as compared with a control group. Furthermore, the antibiotic effect of propolis was evidenced from the collection of material from wound beds, showing that propolis reduced local infection in the wound bed [20].

### 3.2. Surgical Ulcer

Excision of a tumor is a surgical procedure in which a tumor and additional tissue, i.e., the margin, are removed, otherwise there are high recurrence rates due to cancer cells that remain in the tissue. Therefore, the post-surgical period requires the administration of topical compounds to promote healing [24].

A very important study used a formula containing pure propolis in the healing of oncological ulcers after excision. Six patients over 60 years of age, including both sexes, were evaluated and instructed to apply a thin layer of the formulation on the wound with the aid of a spatula, once a day. In view of the results obtained, the minimum time for ulcer healing was 30 days and the maximum was 45 days, with an average of 39 days to close the lesion of the research participants [17].

### 3.3. Venous Ulcers

Venous ulcers represent a health problem due to their high rate of interference in the social and work life of society. The main cause of venous ulcers is venous insufficiency in the lower limbs. Decisions regarding the type of treatment and guidelines for wound prevention require technical and scientific knowledge [25]. A study conducted in 2013 investigated the action of propolis in patients with venous ulcers. In Group 1, 28 patients participated (ulcer area ranging from 6.9 to 9.78 cm^2^). Patients were treated with propolis ointment associated with the use of a short-term compressive bandage. In Group 2, there were 29 patients (ulcer area 7.2–9.4 cm^2^) treated by compression using an Unna’s boot without topical treatment with propolis. On the one hand, the healing time of Group 1 was 6 weeks of treatment. On the other hand, Group 2 had a healing time of 16 weeks. The results show the effectiveness of healing and repair, as well as the faster healing by using propolis ointment as compared with an Unna boot [22].

### 3.4. Wound Due to Sacroccygeal Pilonidal Disease

Sacrococcygeal pilonidal disease is the occurrence of infection in the subcutaneous tissue of the intergluteal groove. The most common target audience includes young male adults. Evidence suggests a higher incidence in individuals with more body hair, those who are obese, those with a deep intergluteal groove, and those with a history of boils elsewhere [26]. One study included 33 patients with sacrococcygeal pilonidal disease wounds after marsupialization who were divided into a control and placebo group. One group was routinely treated with bandages already used in this area and the other group was treated with 15% Anatolian propolis. It is notable that the ulcers had a better evolution in the 7- and 14-day intervals of the postoperative period in the investigational group. The conclusion was that treatment with propolis in these lesions, as long as the lesions were not complicated, accelerated the healing process [21]. Propolis can be used to accelerate wound healing when the marsupialization method is preferred in patients diagnosed with uncomplicated sacrococcygeal pilonidal cysts because of its low cost, good patient compliance, low side effect profile, absence of toxicity, and high efficacy.

### 3.5. Burn Wounds

Mild burns (second degree) are those that are generally classified as superficial, involving only the epidermis and superficial dermis, but sparing dermal skin attachments such as sweat glands and hair follicles. Treatment at this level consists of bandages soaked in saline or equivalent coverage, promoting epithelialization of the lesion [27].

One study compared the effectiveness of propolis versus silver sulfadiazine for treating mild (superficial second degree) burns. Among the selection criteria, participants had bilateral burns of similar size and depth, and each wound received a compound. Initially, the wounds were debrided and cleaned and every 3 days the participants returned to the unit to change the bandage. In short, the antimicrobial effect was not of great significance between the two compounds. However, propolis was shown to be efficient in reducing the inflammatory process and promoting faster healing as compared with silver sulfadiazine [18].

### 3.6. Mouth Ulcers

Since the last century, propolis has been the target of research that evaluates its healing potential in wounds. In 1996, in Cuba, a study was carried out with 78 patients, which evaluated the effects of propolis used in the form of propolin (in an alcoholic vehicle) and propodal (prepared with propylene glycol) on mouth ulcers and in patients treated surgically. A comparison of the results of the groups treated with propolis and the control group that received no treatment, showed that there was an improvement in the healing of mouth ulcers and a faster regression of symptoms [13].

### 3.7. Other Studies

In 1990, Bernardo et al. evaluated the bactericidal and bacteriostatic potential of propolis. Using propolis at a concentration from 3 to 30%, the presence of granulation tissue was observed in patients with chronic ulcers of different etiologies, there was an improvement in the odor (of the secretions), and an improvement in the sensitivity to pain that demonstrated the anesthetic action of propolis. The results of the secretions in culture indicated a decrease in microorganisms, which even became negative [12].

In 2007, Santos and other colleagues conducted a Brazilian study in Paraná, and evaluated the action of a propolis ointment on chronic ulcers in 20 patients. Chronic ulcers were vascular, diabetic, and pressure ulcers. Topical propolis therapy showed improvements in the appearances of the lesions, in the amount of secretion, an increase in granulation tissue, improvement in pain, swelling, and local itching, in addition to an analgesic effect. More than 70% of ulcers healed before the median wound healing time of 13 weeks [9].

Years later, Silva et al. (2017) also investigated propolis ointment for the healing of different types of ulcers (venous, pressure, and diabetic). With the use of propolis ointment at a concentration of 30%, the mean healing time was only 45 days and 20% of the patients had complete wound closure [14].

### 3.8. Propolis Wound Healing Mechanism

The use of propolis extract for treating wounds decreases healing time and accelerates the process of contraction and tissue repair, mainly due to its antimicrobial and anti-inflammatory properties [5].

The specific antimicrobial mechanism of propolis is still unclear, due to the synergistic interaction of the ingredients of propolis, but there are strains that are more susceptible than others to its action [28]. Some studies have suggested that structural damage to the microorganisms is a possible mechanism by which propolis exhibits its antimicrobial activity and this natural substance has multitarget activity in the cell [29]. Propolis acts by inhibiting the replication process of the pathogens and disrupting the ability of the pathogens to invade the host cells by forming a physical barrier and inhibiting enzymes and proteins needed for invasion into the host cells. Furthermore, propolis inhibits the metabolic processes of the pathogens by disrupting cellular organelles and components responsible for energy production [30].

Regarding the anti-inflammatory action, studies have shown that propolis upregulated the innate immunity and modulated the inflammatory signaling pathways [31,32]. Propolis significantly inhibited the lipoxygenase pathway of arachidonic acid metabolism and the expression of the iNOS gene, by acting on the iNOS promoter at the NF-kB site and directly inhibiting the catalytic activity of iNOS. Propolis promoted tumor growth factor β (TGF-β) signal transduction, significantly reduced the levels of matrix metalloproteinases and proinflammatory cytokines and eicosanoids, and enhanced the deposition of type I collagen [33]. In addition, propolis compounds modulated the cellular immune response, suppressing the activation and differentiation of macrophages and the recruitment of leukocytes [34].

## 4. Method

This is a literature review. A bibliographic survey on the clinical use of propolis in skin ulcers was carried out with journals indexed in national and international databases: clinicaltrials.gov, Scielo, Pubmed, Google scholar, Medline and Lilacs, during the period between 1990 and 2021. The keywords, in Portuguese, Spanish, and English, included propolis, healing, skin ulcers, and clinical studies.

## 5. Conclusions

The therapeutic properties of propolis have been demonstrated from the mid-1990s to the present day, mainly through in vitro and in vivo non-clinical studies. Studies on the therapeutic use of propolis in wounds are still scarce, although, since 2002, there has been a notable increase in research. As for the therapeutic action of propolis in wound healing, studies have shown positive results, with a reduction in wound healing time as compared with the controls used. This healing action is due to the antimicrobial, anti-inflammatory, and analgesic/neoangiogenic effects of propolis. Without divergence among the articles, the healing potential of propolis has been evidenced as an alternative treatment for skin wounds such as diabetic, venous, and surgical ulcers, as well as wounds caused by burns or even by sacrococcygeal pilonidal disease. However, there is a need to standardize the type of administration (topical ointment, ethanol extract, spray, aqueous liquid form, etc.) and the concentration of propolis for each type of wound. In addition, further clinical studies are essential to add information about propolis safety and for obtaining the best possible therapeutic benefits from its use, in order to increase scientific evidence, thus, subsidizing new treatment alternatives for people with skin lesions, and also enabling safe and quality care.

## Figures and Tables

**Table 1:** Summary of results about the healing potential of propolis in skin wounds, which has been obtained through clinical studies conducted between 1990 and 2021.

Year	Ref.	Type of Propolis	Goals	Results
2007	[9]	Propolis ointment	To evaluate the evolution of chronic ulcers (vascular, diabetic, and pressure) with topical use of propolis.	The mean wound healing time was 13.1 weeks and the study follow-up was 20 weeks; 74.1% of ulcers healed before 13 weeks. Venous ulcers healed in 35% of patients and pressure ulcers in 10% of patients. Patients reported analgesic effects and improvement of local heat, odor, swelling, secretion, and itching. There was an improvement in the appearance of the lesions, amount of secretion, and an increase in granulation tissue.
1990	[12]	Propolis-water solution (3%) and alcoholic extract (30%)	To describe the biochemical action of propolis and to evaluate its bactericidal and bacteriostatic potential.	The presence of granulation tissue was observed in the patients’ wounds, and there was an improvement in the odor of the secretions and in pain sensitivity, demonstrating the anesthetic action of propolis.
1996	[13]	Propoline (alcoholic vehicle) and propodal (propylene glycol)	To evaluate the effects of propolis in surgical treatments and mouth ulcers.	Tissue recovered after treatment with propolis.
2017	[14]	Propolis ointment (30%)	To analyze the effect of 30% propolis ointment on the healing of different types of ulcers.	The ointment was effective as an alternative healing treatment, with a short healing time, i.e., only 45 days; 20% of patients had complete wound closure.
2019	[15]	Propolis spray (3%) in propylene glycol preparation manufactured by a bee product company in the Maule Region of Chile (Health Authorization no. 639-18 August 2009, Laboratories Rotterdam, Maule, Chile)	To evaluate the effect of propolis as an adjuvant in the healing of human diabetic foot ulcers.	Propolis promoted the closure of a diabetic foot wound and a reduction in the area of the lesion related to an increase in the deposit of extracellular matrix, which aided in healing.
2018	[16]	Propolis ointment (5%), i.e., propolis water extract was added to an ointment base (semi-solid preparations of hydrocarbons such as petrolatum, mineral oil, paraffin, and synthetic hydrocarbons), for a final concentration of 5%	To investigate the effect of topical administration of propolis on the healing of diabetic foot ulcers.	The results of the present study indicated that, although changes in erythema and ulcer secretion were not significantly altered, the area of ulceration was decreased and the wound healing process was improved within 4 weeks.
2012	[17]	Formulation containing propolis, honey, granulated sugar, butter, and powdered albumen	To report the mean healing time of oncological wounds after excision, observed with the aforementioned formulation.	The minimum healing time observed was 30 days and the maximum healing time was 45 days.
2002	[18]	Brazilian propolis ointment	To compare propolis ointment and silver sulfadiazine in mild burns.	Although silver sulfadiazine showed good results, propolis was superior, reducing the inflammatory process and promoting faster healing.
2006	[19]	Propolis (800 mg), myrrh and bee honey (50 mg)	To investigate the effect of a mixture of propolis, myrrh, and bee honey on a diabetic foot ulcer.	Effective healing was observed after four weeks.
2014	[20]	Propolis in watery liquid form, originated from Australia	To determine whether Australian propolis is effective in a pilot study of human diabetic foot ulcer healing.	Ulcer area was reduced by an average of 41% in the propolis group as compared with 16% in the control group at Week 1 and by 63% vs. 44% at Week 3, respectively.
2021	[21]	Anatolian propolis (15%)	To compare the wound healing rate between well-evaluated and standardized use of propolis (Anatolian propolis) and routine bandages in patients with sacrococcygeal pilonidal treated with marsupialization.	Ulcers had better evolutions in the 7- and 14-day intervals of the postoperative period in the investigational group.
2018	[22]	Propolis ointment (7%)	Investigation of the effectiveness of topical treatment of non-healing chronic venous leg ulcers with propolis ointment.	The ulcers healed successfully within the first 6 weeks of treatment using a two-layer bandage and topical application of propolis ointment.

## Data Availability

Not applicable.
